# A Data-Driven Fragmentation Model for Carbon Therapy GPU-Accelerated Monte-Carlo Dose Recalculation

**DOI:** 10.3389/fonc.2022.780784

**Published:** 2022-03-25

**Authors:** Micol De Simoni, Giuseppe Battistoni, Angelica De Gregorio, Patrizia De Maria, Marta Fischetti, Gaia Franciosini, Michela Marafini, Vincenzo Patera, Alessio Sarti, Marco Toppi, Giacomo Traini, Antonio Trigilio, Angelo Schiavi

**Affiliations:** ^1^ Department of Physics, University of Rome “Sapienza”, Rome, Italy; ^2^ INFN (Istituto Nazionale di Fisica Nucleare) section of Roma 1, Rome, Italy; ^3^ INFN (Istituto Nazionale Fisica Nucleare) Section of Milano, Milano, Italy; ^4^ Department of Medico-Surgical Sciences and Biotechnologies, Post-Graduate School in Medical Physics, Rome, Italy; ^5^ Department of Scienze di Base e Applicate per l’Ingegneria (SBAI), University of Rome “Sapienza”, Rome, Italy; ^6^ Museo Storico della Fisica e Centro Studi e Ricerche Enrico Fermi, Rome, Italy; ^7^ INFN Laboratori Nazionali di Frascati, Frascati, Italy

**Keywords:** hadrontherapy, carbon ion (C12), fragmentation, fast MC, quality assurance (QA), graphics processing unit (GPU)

## Abstract

The advent of Graphics Processing Units (GPU) has prompted the development of Monte Carlo (MC) algorithms that can significantly reduce the simulation time with respect to standard MC algorithms based on Central Processing Unit (CPU) hardware. The possibility to evaluate a complete treatment plan within minutes, instead of hours, paves the way for many clinical applications where the time-factor is important. FRED (Fast paRticle thErapy Dose evaluator) is a software that exploits the GPU power to recalculate and optimise ion beam treatment plans. The main goal when developing the FRED physics model was to balance accuracy, calculation time and GPU execution guidelines. Nowadays, FRED is already used as a quality assurance tool in Maastricht and Krakow proton clinical centers and as a research tool in several clinical and research centers across Europe. Lately the core software has been updated including a model of carbon ions interactions with matter. The implementation is phenomenological and based on carbon fragmentation data currently available. The model has been tested against the MC FLUKA software, commonly used in particle therapy, and a good agreement was found. In this paper, the new FRED data-driven model for carbon ion fragmentation will be presented together with the validation tests against the FLUKA MC software. The results will be discussed in the context of FRED clinical applications to ^12^C ions treatment planning.

## 1 Introduction

In Particle therapy (PT) solid tumors are irradiated by means of accelerated charged particle beams (mainly protons and, more recently, carbon ions). The main advantage this technique with respect to the standard radiotherapy using X-rays/electron beams, is related to the different longitudinal energy release profiles. While photons longitudinal dose release is characterized by a slow exponential decrease, for charged particles a sharp peak at the end of the path is observed, providing a more selective energy deposition. By conveniently controlling the Bragg Peak (BP) position by means of the beam energy tuning, it is possible to concentrate the dose to tumors and, at the same time, preserve the surrounding healthy tissues. In carbon ion therapy an important effect that has to be properly accounted for at the treatment planning stage is the contribution to the dose absorption distribution from nuclear fragments produced by the interaction of carbon ions with target nuclei. This process attenuates and mitigates the primary beam contribution while producing secondary fragments with an energy per nucleon comparable to that of the projectile. As a consequence, the total absorbed dose will have a non-negligible contribution due to secondary particles which have different biological effectiveness and range with respect to the primary beam, releasing the dose also in a tail beyond the BP. When comparing carbon ions and protons, it is also important to note that the BP of the former is more resolved and the absorbed dose distribution shows a better ratio between the peak and the plateau region. Another important difference is that photons have a sparse ionization density (low-LET radiation) and protons are considered to the photon-like beside their end-of-range path where they can reach high LET values, while carbon ions are high-LET particles all along their path. The achieved steep, when compared to conventional radiotherapy, dose gradients in PT demand accurate patient positioning and treatment planning to maximize the treatment efficacy. Patient treatment plans are obtained using a Treatment Planning System (TPS) software that provides, accordingly to medical prescriptions, the irradiation details for each particle beam in each field. The commercial TPS used in the clinical routine are mainly based on analytical algorithms that achieve a reduced computation time at the cost of a reduced accuracy in the dose maps calculations. Analytical TPSs have to be routinely tested through quality assurance (QA) tools to verify that the accelerators parameters have been calculated correctly for each patient. In several treatment centers the QA check is performed having the accelerator delivering the beam in a tank full of water following the TPS instructions and then measuring the dose in different target points with several ionization chambers. To improve the analytic TPS usually Monte-Carlo based TPS are employed (e.g. both RayStation and Varian AcurosPT provide full MC support). It has been demonstrated that the use of MC in PT could lead to a significant reduction in treatment planning safety margins (1), thanks to its accurate modelling and calculation of the dose absorbed by the tissues. MC simulations of proton treatment plans have previously been performed using well‐established software packages such as FLUKA (2), GEANT4 and MCNP X (3). Despite the improvements that can be obtained by means of MC dose calculation, pencil-beam-based algorithms are widely used in clinical practice (4), mainly because of their high computational efficiency. On the other hand, the accuracy of a MC dose calculation is determined by the total number of particles used for the simulation, implying that a large number of particles, and long computational times, are needed to yield the desired level of precision. For that reason, the use of full MC simulations, especially in carbon therapy where also the secondary particles emitted need to be accounted for, is limited to the re-calculation of existing treatment plans for research studies, while it is not suitable for a routinely application in the TPS implementation and as QA tool for all patients (5). Despite the great efforts devoted to reducing the MC dose calculation time (6–9), the currently available algorithms and implementations still cannot match the clinical requirements.

The advent of general programming Graphics Processing Units (GPU) has prompted the development of MC algorithms that can significantly reduce the plan recalculation time (10–19) achieving an impressive speed gain compared to CPU‐based calculations, profiting from algorithmic simplifications and hardware acceleration. Exploiting the GPU hardware, many vended TPS used for proton therapy now include MC tools (20–25). For carbon therapy, recently a tool called goCMC (GPU OpenCL Carbon Monte Carlo) (26) was developed.

In this framework, the FRED (Fast paRticle thErapy Dose evaluator) (27) software toolkit has been developed. It is a MC-based software optimized for GPU architecture that has been developed to recalculate and optimize external beams radio therapy treatment plans delivered using either protons, carbon ions, electrons or photons. FRED purpose is to rapidly recalculate a complete treatment plan within minutes, opening the way for many clinical applications where the time-factor is of paramount importance.

## 2 Material and Methods

The FRED core engine has been developed balancing accuracy, calculation time and GPU execution guidelines to achieve the best accuracy in the absorbed dose calculation while exploiting the GPU power to reduce the calculation time. To do so, the most effective physical models from the literature have been chosen, and a careful optimization has been carried out to achieve the needed precision in the dose calculation while avoiding the explicit computation and handling of processes that would result in negligible contributions while affecting the software tracking performance (e.g. atom excitation, the production and tracking of photons, etc.). To reduce the computational time of many physical processes, FRED relies on a library of pre-computed look-up tables. This approach performs extremely well on GPU cards where hardware interpolation can be exploited using the so-called Texture Units. The algorithms core structure is detailed elsewhere (27).

The handling of proton beams interaction with matter implemented within FRED is already at a mature stage, achieving a precision that matches the clinical requirements and allowed its use as a quality assurance tool in the centers of Maastricht and Krakow and as a research tool at several clinical and research centers in Europe (Krakow, Trento, Maastricht, Lyon and PSI). Carbon ion, electron and photon beams have been recently introduced as well for applications in carbon ion therapy, photon radiotherapy and IORT (IntraOperative Radiation Therapy). In this contribution the newly developed data-driven tracking model of carbon ions will be described in detail.

The dose engine for carbon ions relies on three main building blocks that are used to simulate the particles interaction with matter: the ionization energy loss, the multiple scattering and the fragmentation model. The ionization energy loss and multiple scattering implemented in the carbon ions model are analogous to the ones used in FRED for protons (27). For what concerns the multiple coulomb scattering of carbon ion beams, the single Gaussian term included in Highland’s formula (28) to account for such interactions is multiplied by a scaling factor *f_mcs_*, following the approach documented in *Fippel and Soukup* (7). This factor was obtained by comparing FRED and FLUKA simulations of a single pencil beam in water with an energy in the center of the therapeutic range and with nuclear interactions switched off. The values obtained have been computed at different depths, energies and using different ion beams, resulting in values ranging from *f_mcs_* = 1.29 (for 200 MeV/u alpha particles, computed at 15% of range) to *f_mcs_* = 1.43 (for 300 MeV/u oxygen ions, computed at 90% of the range). For each transported charged particle, the best scaling factor was implemented as the one that gave the best lateral distribution of a single pencil beam at the BP placed at a reference depth of 15 cm in water.

The nuclear model, developed completely from scratch, has been parameterized using data already published and this is, as for now, a unique characteristic of FRED. The other available MC software implemented on a GPU hardware that is currently capable of handling carbon ions interactions [goCMC (26)] makes instead use of the information obtained from Geant4 simulations. In particular, for FRED, data used for the calculation of the fragmentation cross sections were extracted from the papers of *Tacheki* (29), *Zhang* (30) and *Kox* (31, 32). Data used for the sampling of the combination of fragments emitted, energy and angle distributions, were taken from the experiments at Ganil (laboratory of CAEN, France), where the fragmentation of carbon ions on thin targets (H, C, O, Al and Ti) has been studied (33, 34). The experiment provided data about the angular and energy cross-section of a carbon beam of 95 and 50 MeV/u and with detection angles in the range (-43°; 43°). To simulate all the energies of interest for carbon ion therapy [namely up to 400 MeV/u as in the case of CNAO center (35)], an algorithm to scale the energy and angle distribution as a function of the incident particle energy has been implemented. Whenever the data were missing, the predictions of the FRED model have been bench-marked instead, against the FLUKA MC.

### 2.1 Nuclear Model

The nuclear interactions of a given particle are handled in two separate steps. First, the probability that a nuclear interaction occurs is computed, taking into account each particle mass attenuation coefficient using the following equation:


(1), $$\frac{\mu }{\rho }=\sum_{i}\frac{N_{A}w_{i}\sigma_{t}^{i}}{A_{i}}$$

where the sum is performed against all the elements of the target compound, *μ* is the attenuation coefficient, *ρ* is the material density, *N_A_* is the number of Avogadro, *w_i_*, *A_i_* and $\sigma_{t}^{i}$ are respectively the mass weight, the atomic mass and the total cross-section of nucleus-nucleus interactions of each i-th element of the target. The total cross-section is defined as the sum of the elastic and non-elastic cross-sections.

Elastic collisions are handled requiring kinetic energy and momentum conservation, and sampling the deflection angle in the center of mass frame. In the case of non-elastic collisions, no energy conservation is implied, the incident carbon ion track is removed from the simulation, and charged fragments are generated by means of a sampling procedure and queued for tracking.

#### 2.1.1 Elastic Cross-Section

The elastic cross-section is explicitly accounted only if the carbon ion projectile interacts with a hydrogen nucleus, as the fragmentation process dominates for all heavier target nuclei. To handle the elastic interactions, we have exploited the center of mass reference system in which the carbon ion interactions with the proton target can be modeled using the data collected studying the reversed process (proton interactions with a carbon ion target).

The sampling of elastic cross-sections was done according to data available from the ENDF database [*ENDF/B-VII Incident-Proton Data* (36)], based on nuclear model calculations benchmarked against experimental data (37, 38).

The relationship between the carbon ion scattering angles, when it interacts with the hydrogen nucleus, in the center of mass and in the laboratory reference frames can be written as:


(2), $$\cos\;(\theta_{l})=\frac{A+\cos\;(\theta_{c})}{\sqrt{A^{2}+2A\cos\;(\theta_{c})+1}}$$

where *θ_l_* and *θ_c_* are respectively the scattering angles in the laboratory and center of mass (CoM) reference frames, A is the atomic mass of the projectile and the atomic mass of the hydrogen has already been considered equal to 1 (39, 40). The carbon ion diffusion angle is hence extracted using, as input, an isotropic distribution computed in the CoM frame.

With the same procedure it is possible to calculate also the proton target deflection (*ϕ_l_*):


(3), $$\cos\;(\phi_{l})=\frac{1+\cos\;(\theta_{c})}{\sqrt{2(1+\cos\;(\theta_{c}))}}$$

The other parameter necessary for the description of the elastic interaction is the new energy of the projectile and of the target element(s). The kinetic energy in the laboratory system after the collision, El' , is:


(4) El'=A2+1+2Acos (θc)(A+1)2El=12[(1+α)+(1−α)cos (θc)]El

where *α* equals to $\frac{{\left(A-1\right)}^{2}}{{\left(A+1\right)}^{2}}$ and *E_l_* is the kinetic energy of the ion in the laboratory system before the collision. Similarly, in the laboratory system the energy of the proton $E_{l}^{p}$ is:


(5) $$E_{l}^{p}=\frac{2AE_{l}}{{\left(A+1\right)}^{2}}(1-\cos\;(\theta_{c}))$$

#### 2.1.2 Non-Elastic Cross-Section

The non-elastic cross-section depends on the crossed material and on the type and energy of incident particle.

**Figure 1:**
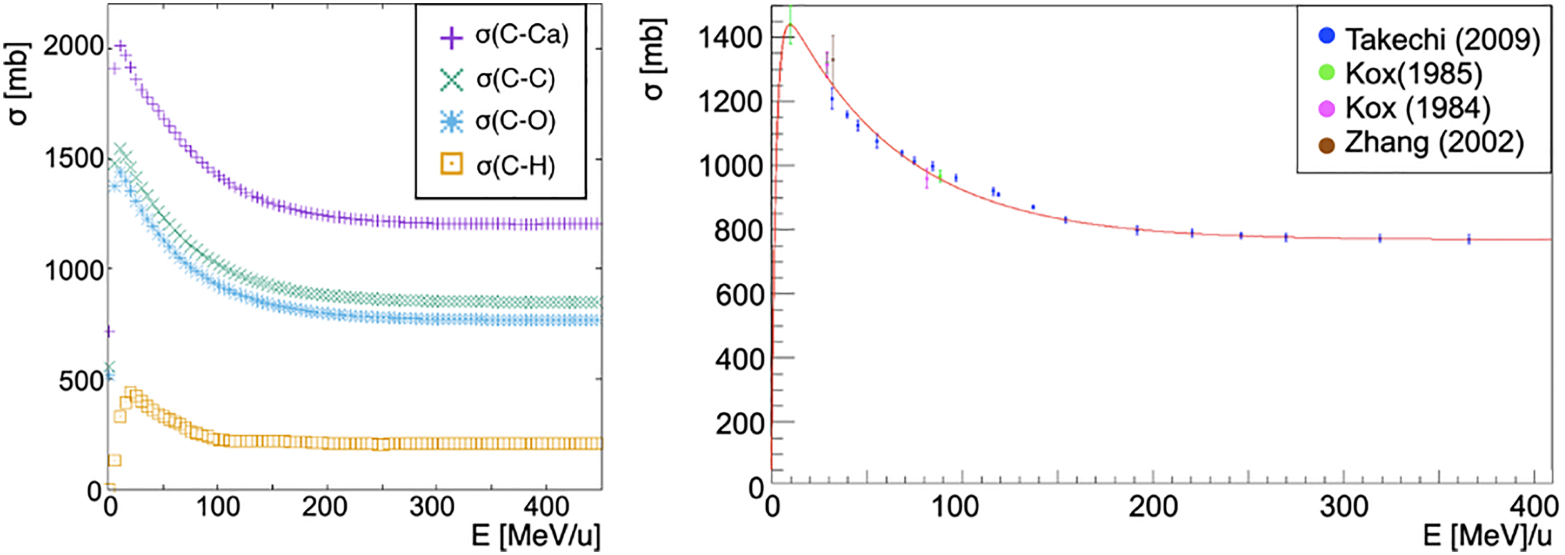
On the left, cross-sections of a carbon ion beam as a function of the energy per nucleon of the projectile interacting with different targets: calcium (purple cross), carbon (green x), oxygen (blue asterisk), hydrogen (orange square). Each cross-section has been obtained as described by Eq. 6 with the exception of the hydrogen target for which the available ICRU data has been used (**Figure 2**). Cross section dependence on the energy per nucleon of the projectile is shown. On the right, fragmentation cross-section in carbon-carbon interactions in the energy range of interest for hadron therapy as a function of the total energy of the projectile. In red the fit to data from papers of *Takechi* (29), Zhang (30) and *Kox* (31, 32).

The cross-section of a nucleus projectile *N_p_* (**Figure 1** left) interacting with a nucleus target *N_t_* is obtained from a fit to existent carbon-carbon interactions data [*Takechi* (29), *Zhang* (30) and *Kox* (31, 32), **Figure 1** right]:


(6) $$\sigma (N_{p},N_{t},E)=K(N_{p},N_{t},E)(1-e^{\frac{-E}{E_{c}}})(p_{0}+p_{1}E+e^{p_{2}-p_{3}E})$$

where *E_c_* = 30 MeV, *p*_0_ = (762 ± 7)mb, *p*_1_ = (14.0 ± 0.7) × 10^–4^ mb MeV^–1^, *p*_2_ = 6.7 ± 0.8 and *p*_3_ = (13.4 ± 0.7) × 10^–3^ MeV^–1^ have been obtained from the fit. *K*(*A_p_*, *A_t_*, *E*) is a scaling factor that is needed whenever the projectile and target nuclei are different from carbon. In particular, the scaling has been obtained using the energy-dependent Kox formula (41–43) for the total cross-section *σ_K_* in nucleus-nucleus reactions


(7) $$K(N_{p},N_{t},E_{cm})=\frac{\sigma_{K}(N_{p},N_{t},E_{cm})}{\sigma_{K}{(}^{12}C{,}^{12}C,E_{cm})}$$

where *σ*_K_(^12^ *C*,^12^ *C*, *E_cm_*) is Kox’s cross-section for carbon on carbon interactions, while *σ_K_* (*N_p_*, *N_t_*, *E_cm_*) is the Kox’s cross-section for a nucleus projectile *N_p_* impinging on a nucleus target *N_t_*.

**Figure 2:**
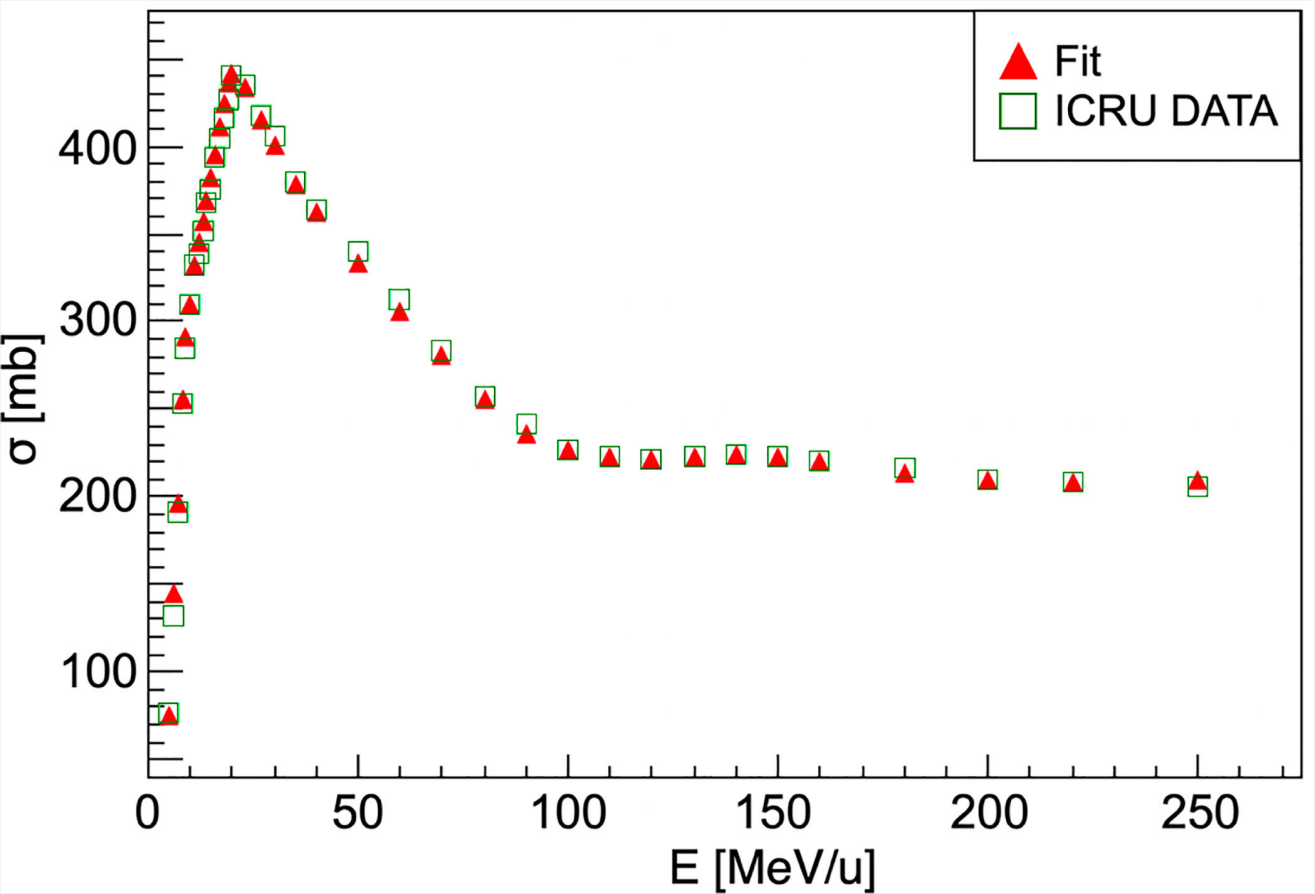
Carbon-hydrogen fragmentation cross-section. For energies higher than 250 MeV/u the cross-section can be considered as nearly constant. Red triangles show the ICRU data fit result that is used in the FRED implementation. ICRU data (41) are represented as green squares.

This scaling law is used for every nucleus of the target except for hydrogen, for which the cross-section has been computed using the available data from ICRU (*International Commission on Radiation Units & Measurements*) (41). The comparison between the available data and the simulation performed using FRED is shown in **Figure 2**. The cross-sections are collected in pre-computed look-up tables that FRED reads and interpolates.

The fragmentation of secondary particles has been computed directly using the Kox formula (42–44). The simulation studies we performed clearly showed that the secondaries fragmentation gives a negligible contribution to the deposited dose. For that reason, by default, the algorithm only takes into account the primary particles fragmentation. We have although left the possibility, for the user interested in evaluating directly the impact of such contribution, to choose whether to enable the accounting for the secondary fragmentation contribution as well.

#### 2.1.3 Fragmentation Model

The nuclear fragmentation process is still lacking a theoretical model capable of providing accurate and precise cross sections predictions. Interactions between the projectile and target nuclei are ultimately described by quantum electrodynamics (QED) but nuclear fragmentation interactions are many-body problems that defy present-day calculation methods at the most fundamental level. The lack of a fundamental theory has been addressed developing a semi-empirical model to describe nucleus-nucleus interactions. The details can be found in the next paragraph.

##### Sampling of the Target

The first step to simulate the fragmentation process in a given medium made of many materials is the choice of the actual nucleus of the target on which the fragmentation occurs. FRED computes such information from tables where a cumulative distribution (explained in detail here-after) is associated with each target used in the 95 MeV/u Ganil experiment and to each possible fragment emitted. To choose which combination of fragments will be emitted, it is necessary to know the nucleus hit by the carbon ion. This information is retrieved using the Kox’s cross-section *σ_K_* (42–44) and computing a cumulative distribution based on the probability for each nucleus to be hit:


(8) $$P^{i}=\frac{n^{i}\sigma_{K}^{i}}{\sigma^{tot}}$$

where


(9) $$\sigma^{tot}=\sum_{j=1}^{j=N}n^{j}\sigma_{K}^{j}$$

and $\sigma_{K}^{tot}$ is the sum of the cross-sections of all the *N* nuclei of the target weighted by the occurrence *n* of each element. For hydrogen and carbon targets, the available cross-section from data is used. As an example, we list the definition of the probability of interaction on hydrogen and oxygen nuclei, *P*(*H*) and *P*(*O*) respectively, for a carbon ion impinging on a water target:


(10) $$P(H)=\frac{2\sigma_{data}^{H}}{\sigma^{tot}},P(O)=\frac{\sigma_{K}^{O}}{\sigma^{tot}}$$

(11) $$\sigma^{tot}=2\sigma_{data}^{H}+\sigma_{K}^{O}$$

where $\sigma_{data}^{H}\text{ and }\sigma_{K}^{O}$ are respectively the cross-sections for a hydrogen target, which is calculated by means of a data fit (**Figure 2**), and the Kox cross-section for an oxygen target. The Kox cross-section is used to compute the probability that an incident particle has an interaction with a given nucleus of crossed material. The algorithm extracts a uniform random number (0,1) to compare with the cumulative distributions in order to choose the element to be used for the fragmentation simulation.

##### Sampling of the Fragments

Once the target nucleus has been determined, the software computes the emission probabilities for each fragment using a look-up table. The isotopes with a non-negligible production cross section are: neutrons, ^1^H, ^2^H, ^3^H, ^3^He, ^4^He, ^6^He, ^6^Li, ^7^Li, ^7^Be, ^9^Be, ^10^Be, ^8^B, ^10^B, ^11^B, ^10^C, ^11^C and ^12^C. The probability table has been computed using an iterative algorithm [*Newton Method* (45)] that allowed to reach a good agreement with the data published from the 95 MeV/u Ganil experiment. This iterative procedure was implemented to account for the experimental correlation between the different fragments production probabilities measured by Ganil. **Table 1** reports the results of the procedure, used for all the targets with the exception of hydrogen, for each isotope and elemental target in comparison with Ganil experiment measurements.

**Table 1:** Production probabilities per isotope and for each elemental target reported in the Ganil experiment (33) and built for the code FRED.

	Probabilities [%]					
Frag	(Ganil) H	(Fred) H	(Ganil) C	(Fred) C	(Ganil) O	(Fred) O
n	–	1.4x10	–	6.5x10	–	6.0x10
^1^H	52(8)	3.8x10	35(2)	1.0×10	38(4)	1.6×10
^2^H	9(2)	5.0	16.3(0.8)	7.5	17(1)	8.8
^3^H	2.0(0.4)	1.3	6.6(0.4)	6.1	6.5(0.7)	5.1
^3^He	5.2(0.5)	3.0	7(1)	1.2	7.2(0.9)	1.7
^4^He	25(10)	26	25(6)	6.4	22(7)	6.3
^6^He	1.3(0.1)	3.6×10^-2^	1.0(0.2)	1.7	1.0(0.4)	1.0
^6^Li	1.5(0.8)	2.3	1.4(0.2)	2.5×10^-1^	1.3(0.3)	2.8×10^-1^
^7^Li	1.0(0.2)	9.3×10^-1^	1.2(0.2)	4.1×10^-1^	1.2(0.3)	3.9×10^-1^
^7^Be	2.0(0.4)	1.6	1.0(0.2)	8.3×10^-2^	1.0(0.2)	1.2×10^-1^
^9^Be	–	2.5×10^-1^	4(1)×10^-1^	1.1×10^-1^	3.4(0.7)×10^-1^	7.9×10^-2^
^10^Be	–	1.0×10^-4^	1.8(0.4)×10^-1^	2.4×10^-1^	1.9(0.5)×10^-1^	1.0×10^-1^
^8^B	–	1.5×10^-1^	1.3(0.4)×10^-1^	1.3×10^-2^	1.2(0.5)×10^-1^	1.4×10^-2^
^10^B	–	1.3	10(3)×10^-1^	8.9×10^-2^	9(6)×10^-1^	8.6×10^-2^
^11^B	–	2.1	1.2(0.5)	2.0×10^-1^	1(1)	1.8×10^-1^
^10^C	–	1.9×10^-1^	1.7(0.6)×10^-1^	1.7×10^-2^	1.5(0.9)×10^-1^	1.6×10^-2^
^11^C	–	3.9	1.1(0.4)	5.5×10^-2^	1.0(0.7)	7.1×10^-2^
^12^C	–	5.9×10^-1^	1.6(0.9)	4.3×10^-2^	1.5(0.9)	7.9×10^-2^

Since the Ganil experiment, as shown in **Table 1**, did not had any experimental access to the production of fragments heavier than ^7^Be in the case of a hydrogen target, in that case the algorithm uses as input the cumulative distributions obtained from a FLUKA simulation^1^ of the interactions of a 95 MeV/n carbon beam impinging on a thin target. The same holds also for the neutron production, absent in the Ganil data.

Outgoing particles from a heavy-ion fragmentation reaction are typically described as either “projectile” or “target” fragments. In the Ganil experiment, both types of fragments were detected and it was impossible to distinguish them. For that reason, both phenomena were considered to be present when using the cumulative distributions. The fragmentation production probabilities were also scaled to account for the non-negligible contribution from elastic scattering of ^12^C isotopes. The simulation of each event proceeds using random numbers to sample, by means of the cumulative distributions previously described, the projectile fragments. The same procedure is used for the target fragmentation.

Once the complete set of fragments is defined, the energy and angle computation are obtained as described in the following section. If the sum of the energy of all projectile’s and target’s fragments is greater than the energy of the projectile, the software extracts a new set of fragments until mass, charge and energy are conserved. The most frequent fragments are neutrons, protons, deuterium and Helium-4 followed by lighter fragments.

##### Sampling of Energy and Angular Distributions

**Figure 3:**
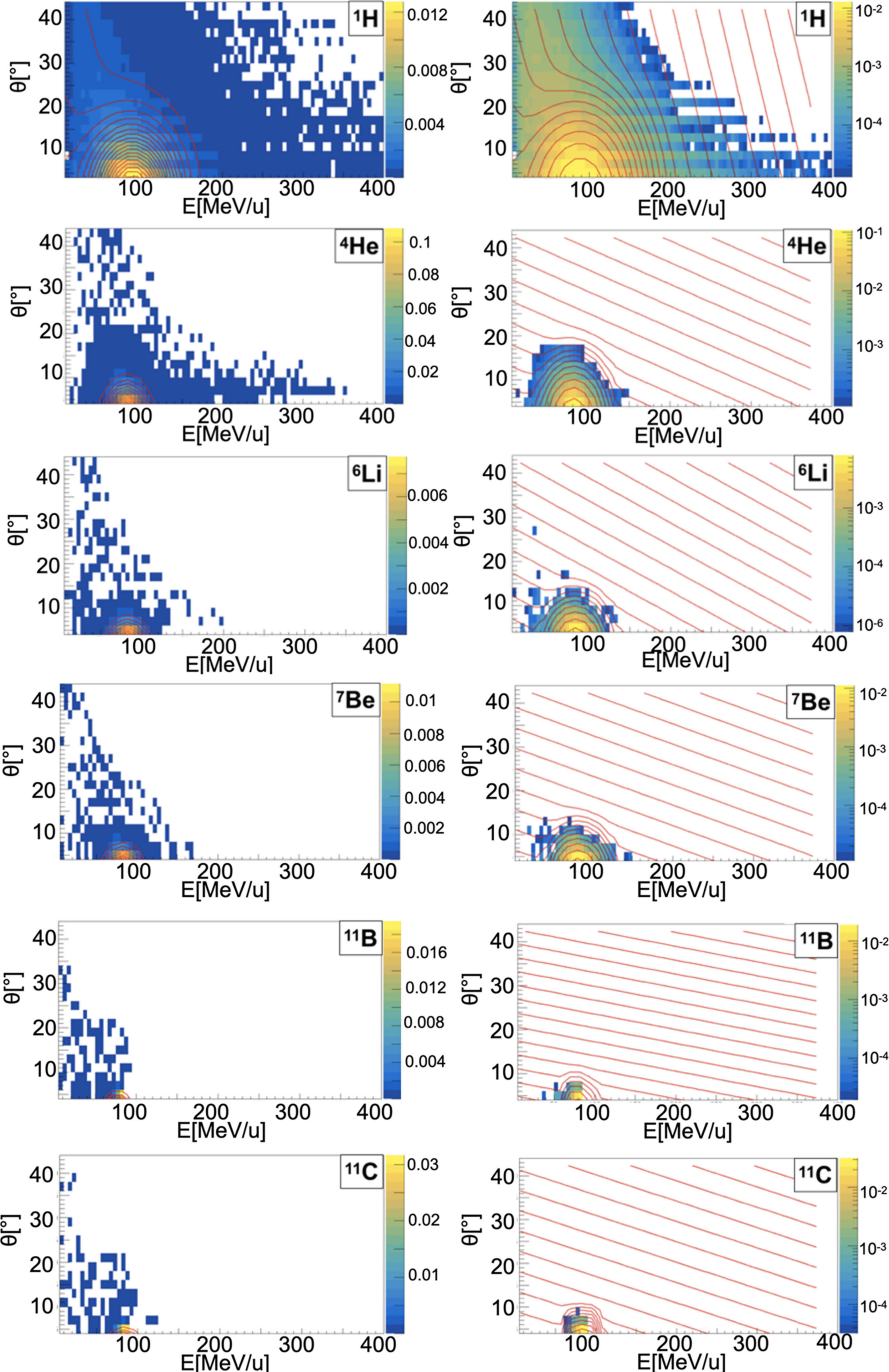
Contour lines (red) of bidimensional fits of energy and angle distribution of different fragments produced by a 95 MeV/u carbon ion beam interacting with a hydrogen target. The color maps represent data taken from the 95 MeV/u Ganil experiment in linear (left) and logarithmic (right) scale.

**Figure 4:**
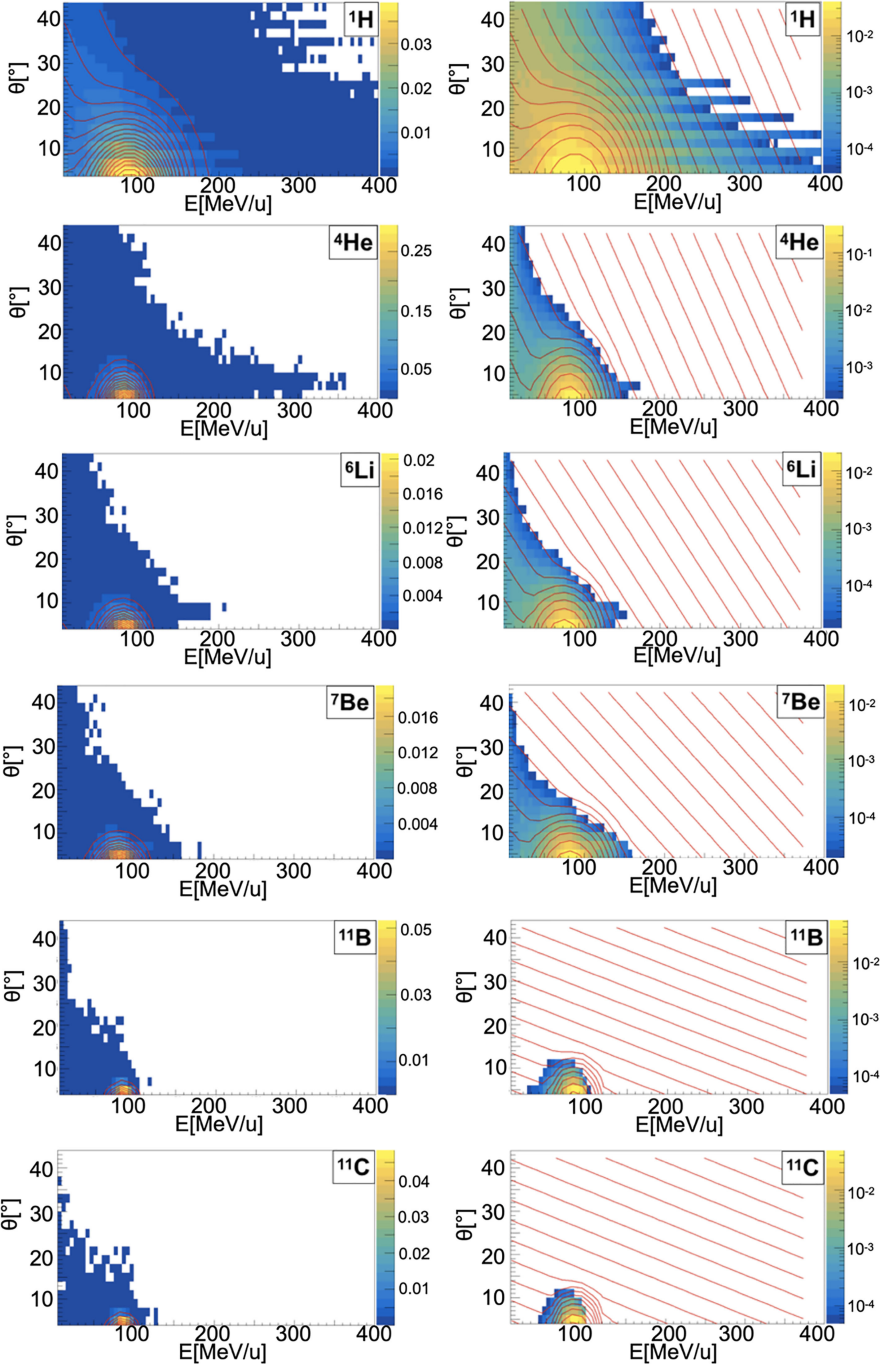
Contour lines (red) of bidimensional fits of energy and angle distribution of different fragments produced by a 95 MeV/u carbon ion beam interacting with a carbon target. The color maps represent data taken from the 95 MeV/u Ganil experiment in linear (left) and logarithmic (right) scale.

**Figure 5:**
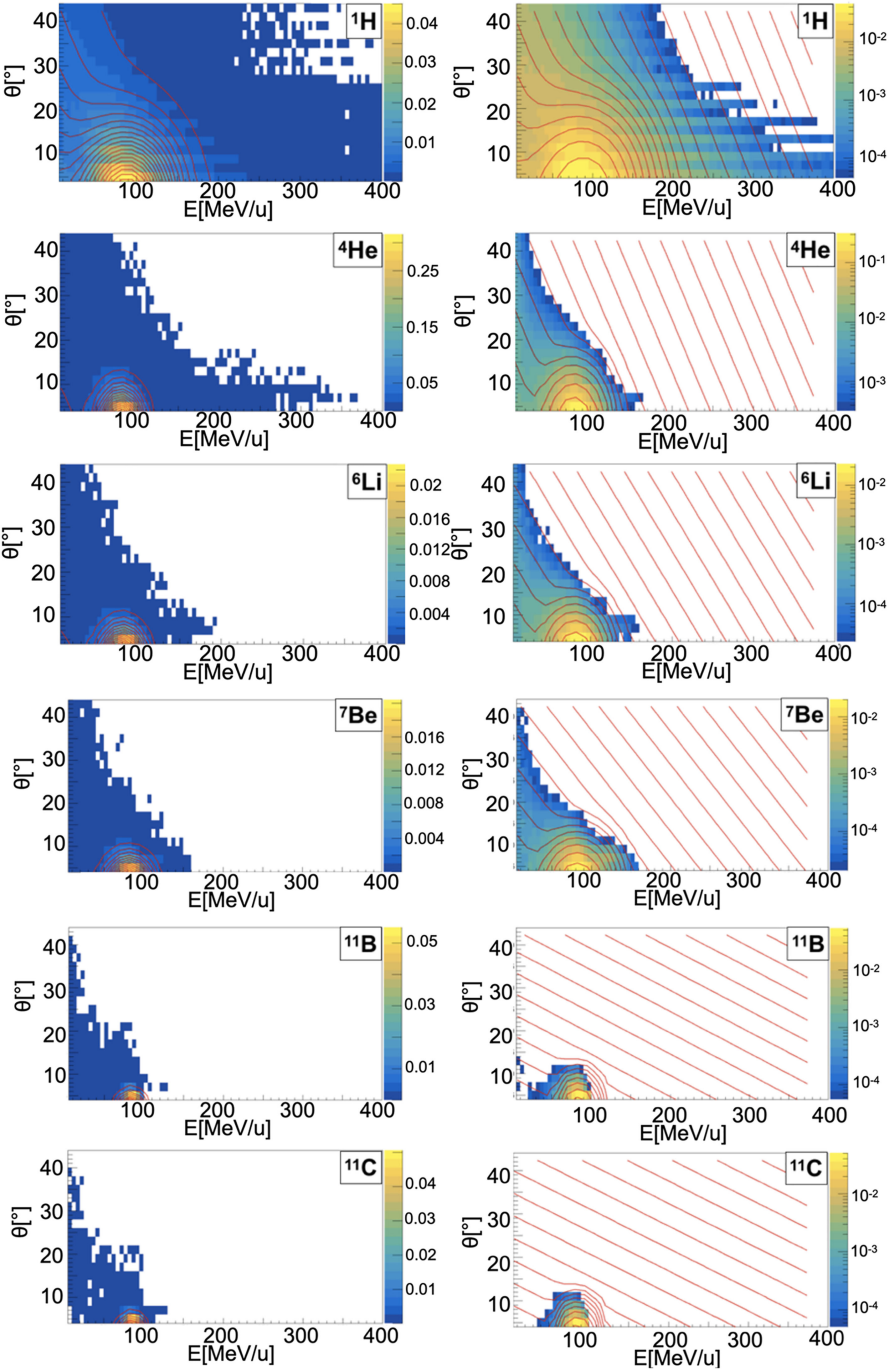
Contour lines (red) of bidimensional fits of energy and angle distribution of different fragments produced by a 95 MeV/u carbon ion beam interacting with an oxygen target. The color maps represent data taken from the 95 MeV/u Ganil experiment in linear (left) and logarithmic (right) scale.

When a projectile particle with a velocity *v* interacts with a fixed target, the produced projectile and target fragments have different angular and energy distributions. While projectile fragments are emitted mostly forward (small angles of emission) and have, on average, the same energy per nucleon of the projectile, target fragments have lower energies and their space distribution is more isotropic. *Golovkov and Matsufuji* (46, 47) observed that, to describe the energy and angular distributions of secondary fragments, Gaussian and exponential distributions are needed. The first one accounts for fragments produced by the projectile, while the latter one for the target fragmentation. In Ganil data the two contributions were mixed and could not be disentangled. In **Figures 3**–**5**, it is possible to observe an example of energy and angle distributions, in linear and logarithmic scales, for the six different fragments (^1^H, ^4^He, ^6^Li, ^7^Be, ^11^B and ^11^C) detected by the 95 MeV/u Ganil experiment after the interaction of a ^12^C ion beam with hydrogen, carbon and oxygen targets respectively.

The measured fragment angles were in the range between 4° and 43°, while the applied energy threshold was fragment-dependent (ranging from 4 MeV for ^1^H to 86.9 MeV for ^12^C). The bi-dimensional phenomenological distribution, *f(E, θ)*, built using a combination of Gaussian and exponential functions, which better describes the data is the following:


(12), $$f(E,\theta )=A_{1}e^{\alpha_{E}E+\alpha_{\theta }\theta }+A_{2}e^{-\left(\frac{{\left(E-<E>\right)}^{2}}{2\sigma_{E}}+\frac{{\left(\theta -<\theta >\right)}^{2}}{2\sigma_{\theta }}\right)}$$

where A_1_, A_2_, *α_E_*, *α_θ_*, *σ_E_*, *σ_θ_*, < *E* > and < θ > are respectively the different normalization factors of the projectile and target contributions, the empirical coefficients that take into account the target fragments energy and angle dependency and the mean and spread, in energy and angle, values describing the projectile fragments distributions. The parameters used in FRED are shown in **Tables 2**–**4** for hydrogen, carbon and oxygen target respectively.

**Table 2:** Parameters of Eq. 12 considering a 95 MeV/u carbon ion beams on a ^1^H target.

Frag.	*A_1_*	*A_2_*	*< E >*	*σ_E_*	*< θ >*	*σ_θ_*	*α_E_*	*α_θ_*
n	5.4×10^-1^	4.9×10	9.3×10	3.3×10	0.0	9.7	1.0×10^-2^	2.5×10^-2^
^1^H	5.4×10^-1^	4.9×10	9.3×10	3.3×10	0.0	9.7	1.2×10^-2^	2.5×10^-2^
^2^H	2.0×10^-1^	7.5	8.1×10	2.3×10	0.0	8.2	2.2×10^-2^	6.5×10^-2^
^3^H	7.8×10^-2^	2.7	7.6×10	2.2×10	0.0	6.8	1.9×10^-2^	1.8×10^-1^
^3^He	2.2×10^-2^	7.6	9.6×10	2.7×10	0.0	6.2	1.8×10^-2^	1.0×10^-1^
^4^He	9.8×10^-3^	5.6×10	8.4×10	1.2×10	0.0	4.3	1.5×10^-2^	1.8×10^-1^
^6^He	3.0×10^-2^	3.1	7.8×10	1.4×10	0.0	4.0	2.6×10^-2^	2.7×10^-1^
^6^Li	8.0×10^-3^	4.5	8.4×10	1.1×10	0.0	3.4	1.9×10^-2^	2.0×10^-1^
^7^Li	1.9×10^-2^	3.3	7.9×10	8.6	0.0	3.2	2.0×10^-2^	2.7×10^-1^
^7^Be	3.6×10^-3^	6.6	8.5×10	1.1×10	0.0	3.1	1.7×10^-2^	2.1×10^-1^
^9^Be	2.0×10^-2^	1.3	8.2×10	7.3	0.0	3.0	2.3×10^-2^	2.7×10^-1^
^10^Be	7.1×10^-2^	3.8×10^-1^	7.9×10	5.3	0.0	3.1	2.4×10^-2^	3.2×10^-1^
^8^B	6.3×10^-1^	8.3×10^-3^	8.9×10	1.3×10	0.0	3.2	2.2×10^-2^	1.6×10^-1^
^10^B	3.5×10^-3^	1.3×10	8.3×10	6.5	0.0	2.5	1.9×10^-2^	3.7×10^-1^
^11^B	1.1×10^-2^	1.4	8.3×10	4.6	0.0	2.2	1.8×10^-2^	5.8×10^-1^
^10^C	1.2×10^-3^	3.7	8.8×10	7.2	0.0	2.3	1.8×10^-2^	2.1×10^-1^
^11^C	5.0×10^-4^	3.9×10	8.4×10	4.8	0.0	2.1	1.7×10^-2^	3.0×10^-1^
^12^C	5.0×10^-4^	9.3×10	8.3×10	3.6	0.0	9.2×10^-1^	1.0×10^-2^	2.0×10^-1^

Parameters relative to the energy, ***E***, are expressed in MeV/u while the one of the angle, θ, in degrees.

**Table 3:** Parameters of Eq. 12 considering a 95 MeV/u carbon ion beams on a ^12^C target.

Frag.	*A_1_*	*A_2_*	< *E* >	*σ_E_*	< θ >	*α_θ_*	*α_E_*	*α_θ_*
n	2.8×10^-1^	1.0×10^2^	9.3×10	3.7×10	0.0	1.0×10	1.2×10^-2^	2.5×10^-2^
^1^H	2.8×10^-1^	1.0×10^2^	9.3×10	3.7×10	0.0	1.0×10	1.3×10^-3^	2.6×10^-2^
^2^H	2.7×10^-1^	5.4×10	8.1×10	2.6×10	0.0	8.9	2.6×10^-2^	3.1×10^-2^
^3^H	2.6×10^-1^	2.5×10	7.3×10	1.8×10	0.0	7.6	3.2×10^-2^	5.7×10^-2^
^3^He	1.5×10^-1^	3.6×10	9.2×10	2.9×10	0.0	7.0	3.1×10^-2^	4.7×10^-2^
^4^He	7.4×10^-2^	1.9×10^2^	8.3×10	1.5×10	0.0	5.2	2.9×10^-2^	8.0×10^-2^
^6^He	8.1×10^-2^	1.0×10	7.8×10	1.7×10	0.0	5.4	3.0×10^-2^	1.5×10^-1^
^6^Li	7.0×10^-2^	1.3×10	8.4×10	1.4×10	0.0	4.4	2.7×10^-2^	1.1×10^-1^
^7^Li	6.1×10^-2^	1.3×10	7.9×10	1.3×10	0.0	4.2	3.1×10^-2^	1.2×10^-1^
^7^Be	3.7×10^-2^	1.2×10	8.3×10	1.6×10	0.0	4.2	2.6×10^-2^	1.2×10^-1^
^9^Be	3.6×10^-2^	5.7	8.3×10	1.1×10	0.0	3.7	2.5×10^-2^	2.2×10^-1^
^10^Be	4.8×10^-2^	3.0	8.2×10	9.3	0.0	3.6	2.4×10^-2^	2.7×10^-1^
^8^B	1.8×10^-2^	1.9	8.8×10	1.7×10	0.0	4.0	2.8×10^-2^	1.5×10^-1^
^10^B	7.7×10^-3^	1.9×10	8.6×10	9.3	0.0	3.2	2.3×10^-2^	2.1×10^-1^
^11^B	8.4×10^-3^	3.9×10	8.4×10	7.3	0.0	2.9	2.0×10^-1^	3.0×10^-1^
^10^C	5.9×10^-3^	3.5	8.8×10	9.5	0.0	3.1	1.9×10^-2^	2.1×10^-1^
^11^C	3.4×10^-3^	3.0	8.6×10	7.2	0.0	2.7	1.9×10^-2^	2.6×10^-1^
^12^C	3.5×10^-3^	6.5×10	8.8×10	4.9	0.0	2.3	1.6×10^-2^	2.8×10^-1^

Parameters relative to the energy, ***E***, are expressed in MeV/u while the one of the angle, ***θ***, in degrees.

**Table 4:** Parameters of Eq. 12 considering a 95 MeV/u carbon ion beams on a ^16^O target.

Frag.	*A_1_*	*A_2_*	< *E* >	*σ_E_*	< θ >	*α_θ_*	*α_E_*	*α_θ_*
n	3.0×10^-1^	1.3×10^2^	9.3×10	3.7×10	0.0	1.0×10	1.3×10^-2^	2.4×10^-2^
^1^H	3.0×10^-1^	1.3×10^2^	9.3×10	3.7×10	0.0	1.0×10	1.3×10^-2^	2.4×10^-2^
^2^H	3.0×10^-1^	6.3×10	8.2×10	2.6×10	0.0	9.2	2.6×10^-2^	3.0×10^-2^
^3^H	2.6×10^-1^	2.8×10	7.3×10	1.8×10	0.0	7.9	3.2×10^-2^	5.6×10^-2^
^3^He	1.5×10^-1^	4.2×10	9.1×10	2.9×10	0.0	7.3	2.9×10^-2^	4.3×10^-2^
^4^He	8.3×10^-2^	2.1×10^2^	8.3×10	1.5×10	0.0	5.3	2.9×10^-2^	7.8×10^-2^
^6^He	8.1×10^-2^	1.1×10	7.9×10	1.7×10	0.0	5.5	2.9×10^-2^	1.4×10^-1^
^6^Li	7.9×10^-2^	1.5×10	8.4×10	1.4×10	0.0	4.5	2.8×10^-2^	1.0×10^-1^
^7^Li	6.3×10^-2^	1.4×10	7.9×10	1.3×10	0.0	4.4	3.1×10^-2^	1.1×10^-1^
^7^Be	3.9×10^-2^	1.3×10	8.3×10	1.6×10	0.0	4.3	2.7×10^-2^	1.1×10^-1^
^9^Be	3.1×10^-2^	6.0	8.3×10	1.2×10	0.0	3.9	2.7×10^-2^	1.8×10^-1^
^10^Be	5.5×10^-2^	3.2	8.2×10	8.8	0.0	3.7	2.4×10^-2^	2.4×10^-1^
^8^B	2.2×10^-2^	2.1	8.9×10	1.7×10	0.0	4.1	2.9×10^-2^	1.4×10^-1^
^10^B	7.7×10^-3^	1.9×10	8.4×10	9.3	0.0	3.3	2.6×10^-2^	1.7×10^-1^
^11^B	8.0×10^-3^	3.1×10	8.5×10	7.1	0.0	2.9	2.1×10^-2^	2.5×10^-1^
^10^C	8.8×10^-3^	3.5	8.8×10	9.2	0.0	3.2	2.3×10^-2^	2.0×10^-1^
^11^C	3.4×10^-3^	3.0×10	8.6×10	7.1	0.0	2.8	2.0×10^-2^	2.3×10^-1^
^12^C	3.4×10^-3^	6.2×10	8.7×10	4.7	0.0	2.4	1.7×10^-2^	3.0×10^-1^

Parameters relative to the energy, ***E***, are expressed in MeV/u while the one of the angle, ***θ***, in degrees.

The projectile fragments have an average energy per nucleon that is close to the projectile one (in this case 95 MeV/u) and their direction is peaked at zero degrees along the incoming beam direction. The target fragments angular distribution is instead almost isotropic and the energy is smaller than the energy of the projectile fragments. The contribution of the projectile fragments term becomes more important when the fragments are heavier. For the hydrogen fragments (^1^H, ^2^H and ^3^H) the energy and angle of emission are extracted directly by Eq. 12 both for projectile and target fragmentation.

The reason of this choice is that, as it can be observed in **Figures 3**–**5**, the Gaussian and exponential distributions for these fragments are largely overlapping and they are not easily distinguishable. All other fragments are extracted from the Gaussian and the exponential distribution in case of projectile and target fragmentation respectively. For a hydrogen target (**Figure 3** and **Table 2**), with the exception of ^1^H fragments, all the distributions have a predominant Gaussian component. This is because the target fragmentation can only produce a proton. The small exponential contribution can be explained as the cross-sections for the hydrogen target have been obtained by subtraction using the cross-sections of CH_2_ and C targets. The distribution fitted in the experimental angular range [4°; 43°] is used to perform an extrapolation to cover the full [0°;180°] range in the angular sampling.

##### Extrapolation to Different Beam Energies

The angle and energy distributions collected by the Ganil experiment correspond to fragments produced by a carbon beam of 95 MeV/u. To consider every possible energy of the projectile, a scaling model has been implemented.

When sampling the projectile fragmentation, the emission energy per nucleon of the i-th fragment is scaled according to the following equation:


(13) $$E^{i}[\text{MeV/u}]=E_{95\text{MeV/u}}^{i}\frac{E_{proj}[MeV/u]}{95[MeV/u]}(1-k)$$

where $E_{95\text{MeV/u}}^{i}$ is the energy per nucleon extracted from the Gaussian distribution of the Ganil experiment and *E_proj_* is the energy per nucleon of the projectile. *k* is used to take into account that the fragments energy from the same event is correlated and that the total energy must not exceed the energy of the projectile:


(14) k=c(1−R)

where *c* is the correlation factor and *R*, for each i-th fragment, depends on the energy of the previous i-1 fragments:


(15) $$R=\frac{E_{nucl}^{i}}{E_{p}}$$

(16) $$E_{nucl}^{i}=\frac{\Sigma_{j=0}^{j=i}E_{j}A_{j}}{\Sigma_{j=0}^{j=i}A_{j}}$$

*E_j_* and *A_j_* are the energy and the atomic number of the previous fragments in the current event.

For the results shown in this paper, the value implemented (c=0.4) was chosen in order to achieve the best agreement between FRED and FLUKA results.

With this linear dependence, the assumption that the average energy per nucleon of a projectile fragment is the same as that of the projectile itself is guaranteed. The sampling of the energy released to the target fragments is analogous to the one of the projectile but $E_{95\text{Mev/u}}^{i}$ is extracted from the exponential distribution without any correlation factors.

The scaling factor for the angle of emission (*θ*) of the projectile fragment, can be computed according to:


(17) $$\left|\vec{p}\right|\sin(\theta )=p_{⊥}$$

where $\left|\vec{p}\right|$ and $p_{⊥}$ are the fragment momentum magnitude and transverse momentum respectively and θ is the angle of $\vec{p}$ with respect to projectile direction. As the angles of emission of projectile fragments are small, it is possible to write:


(18) $$\theta ∼\sin(\theta )=\frac{p_{⊥}}{\left|\vec{p}\right|}$$

The fragment transverse momentum does not depend on the projectile energy. As a consequence, the dependence of the angle on the beam energy is only due to the denominator of Eq. 18:


(19) $$\frac{\theta }{\theta_{95\text{MeV/}u}}=\frac{\left|{\vec{p}}_{95\text{MeV/}u}\right|}{\left|\vec{p}\right|}$$

where *θ_95MeV/u_* is the angle extracted from the Gaussian distribution of the Ganil experiment and $\left|{\vec{p}}_{95\text{MeV/u}}\right|$ is the corresponding momentum.

At therapeutic energies, $p\propto \sqrt{E}$ and hence the equation becomes:


(20) $$\frac{\theta }{\theta_{95\text{MeV/}u}}=\frac{\sqrt{E_{95\text{MeV/}u}}}{\sqrt{E}}$$

where *E_95MeV/u_* and *E* are the fragments kinetic energies of the Ganil experiment and of the fragments emitted for a generic beam energy. Using the fragments energy scaling factor (Eq. 13), the relation between an angle of emission *θ* produced by a projectile of energy *E_proj_* and the angle of Ganil data, $\theta_{95MeV/u}^{i}$ , becomes


(21) $$\theta^{i}=\theta_{95\text{MeV/u}}^{i}\sqrt{\frac{95}{E_{proj}[MeV/u]}}$$

This scaling is not used for protons and neutrons since, checking the angular dependence with FLUKA, it has been observed that for those particles at the energies of interest for particle therapy applications the angle of emission is nearly energy-independent. The same scaling factor is also used for the angle of the target fragments.

## 3 Results

The nuclear models implemented in FRED were tested against the results obtained with a full-MC simulation performed using FLUKA. In particular, the longitudinal and lateral dose distribution obtained simulating the interactions of different beams with different targets have been compared in several configurations and projectiles. In this contribution, we report in detail the results obtained studying the carbon ions beam interactions with a water target and with a patient CT.

### 3.1 Single Pencil-Beam in Water

**Figure 6:**
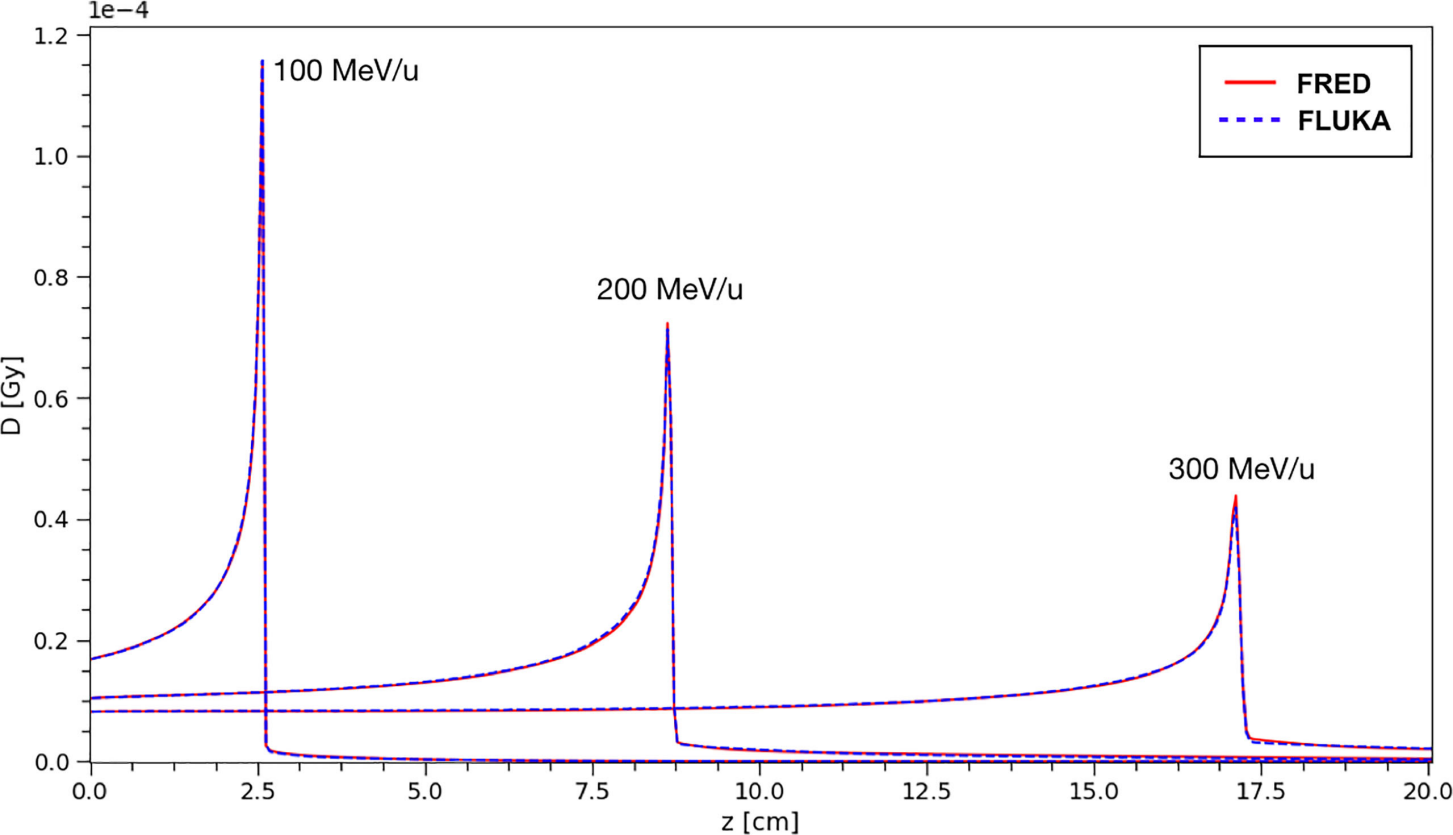
The absorbed dose integrated over the longitudinal axis for carbon ion beams in water at different energies. The absorbed dose per primary particle was obtained simulationg 10^8^ primaries. Comparison between FRED (red continuous line) and FLUKA (blue dotted line) simulations, with the same scoring grid, and the same number of primaries is presented.

**Figure 6** shows the depth-dose profiles obtained from a simulation performed using FRED, in which carbon ions with energies in the range of interest for PT applications (100-300 MeV/u) are interacting with a water target. The target dimensions are 10 cm × 10 cm × 40 cm (*x* × *y* × *z*) with a voxel size of 0.5 mm in all the directions. The incident beam in all the cases was directed along *z*.

The same distributions have been obtained using a FLUKA simulation and the results have been compared. In particular, the curves shown in **Figure 6** correspond to single pencil-beams of mono-energetic carbon ions.

The absorbed dose per primary is shown, from a simulation performed using 10^8^ primary ions to minimize the statistical fluctuations. The profiles closely overlap and, in particular, the agreement of the absorbed dose at the peak between FRED and FLUKA simulations is very good. The relative difference between FRED and FLUKA predictions is always within 2.5% when computing the integral absorbed dose over the whole depth in the 100-300 MeV/u energy range, with the best agreement achieved at 100 MeV/u (relative difference = 0.05%).

**Figure 7:**
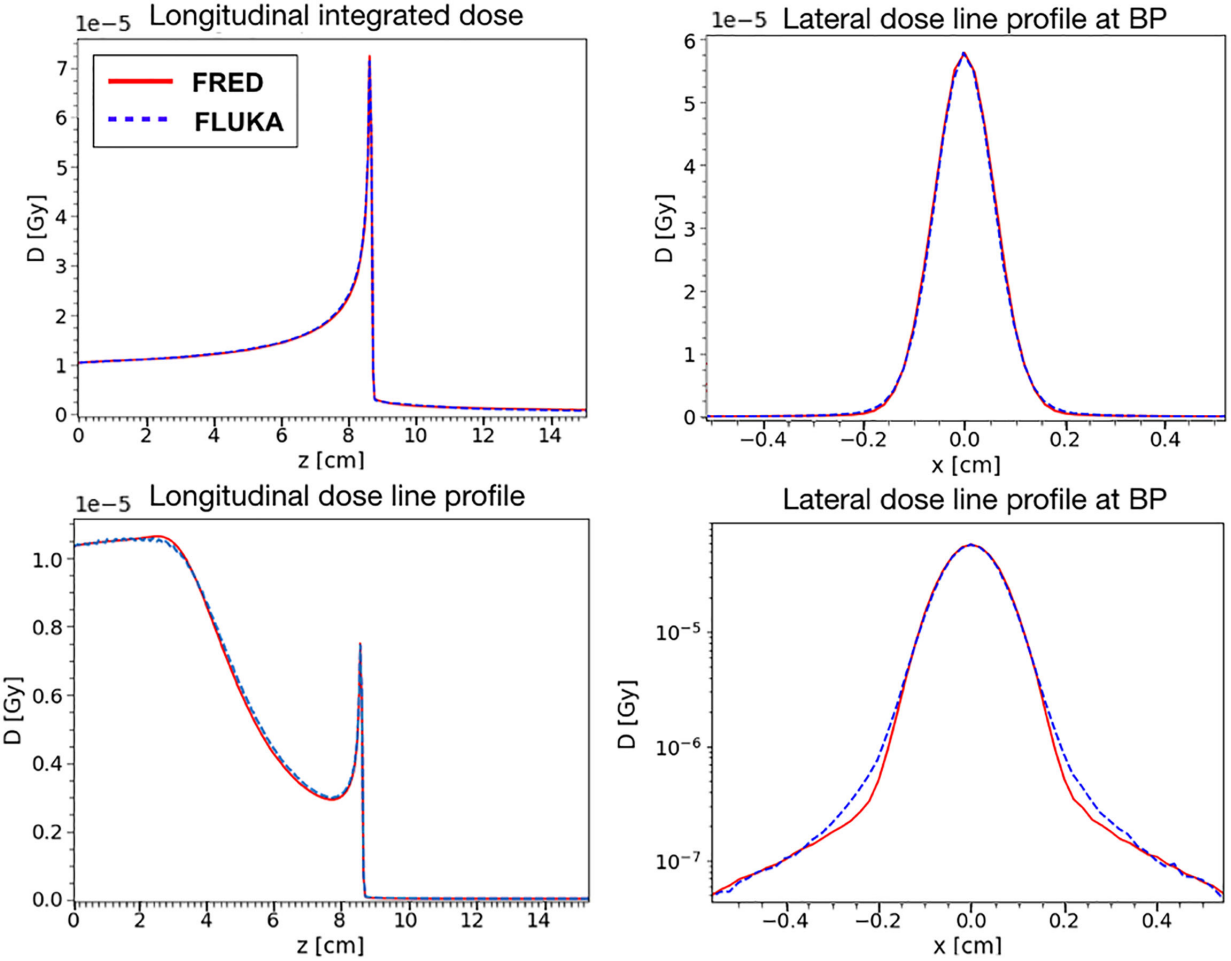
Absorbed dose in water for a 200 MeV/u carbon ion beam simulated with FRED (red continuous line) and FLUKA (blue dotted line) with the same scoring grid and the same number of primaries. On the left, it is possible to observe the absorbed dose integrated over the longitudinal axis (top) and central axis profile along beam axis (bottom). On the right, the lateral axis profile at 8.6 cm of depth in linear scale (top right) and logarithmic (bottom right) scale. This position is the one corresponding to the maximum value of the dose (BP) both in the FLUKA and the FRED simulations.

The agreement between FRED and FLUKA, studied using the same scoring grid and the same number of primaries, is shown in **Figure 7**. A single pencil beam of 200 MeV/u has been simulated along the beam axis (longitudinal) and at the BP position (lateral).

The position chosen for the BP corresponds to the maximum of the dose observed in FLUKA and FRED simulations. With the same scoring grid, the two simulations predict the BP in the same voxel. The lateral transverse profiles show, in linear and logarithmic scale, the tails of the distribution, mainly due to nuclear interactions. Observing the lateral and longitudinal profiles, we can conclude that the present implementation of multiple Coulomb scattering, of nuclear elastic scattering and the angular distribution of secondary fragments are capable of reproducing the main features of the dose distribution.

### 3.2 SOBP in Water

After having studied the dose released by a single pencil beam, the next step was to assess agreement also for a Spread-out Bragg Peak (SOBP) composed by pencil-beams of different energies.

This is a more interesting benchmark considering the purpose of the software. In particular, we have simulated a SOBP corresponding to a 5 cm cuboid starting at a depth of 10 cm with ~2 Gy of physical dose in the center. It has been simulated in a water phantom of dimensions 5 cm × 5 cm × 20 cm (*x* × *y* × *z*) and with a voxel size of 0.5 × 0.5 × 0.2 mm^3^ both with FLUKA and FRED. The incident beams were along z direction.

**Figure 8:**
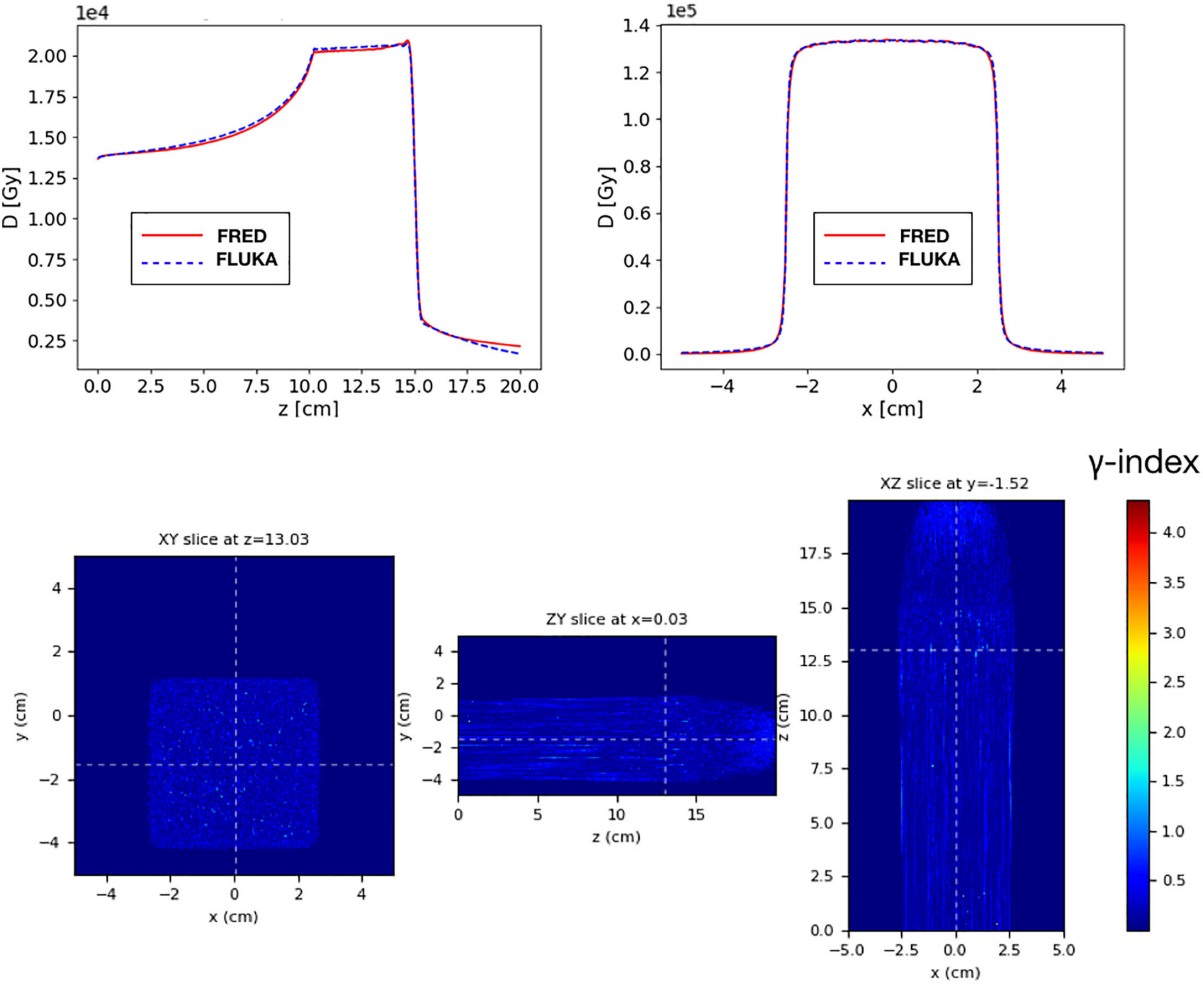
Top: longitudinal (left) and lateral (right) integrated dose distributions for a SOBP in water. FRED (red continuous line) and FLUKA (blue dotted line) simulations are shown using the same scoring grid (voxel size: 0.5 × 0.5 × 0.2 mm^3^), and the same number of primary particles (10^8^). Bottom: the corresponding γ- index distribution is shown. The γ-index 2mm/3% pass rate is 99.89%. The maximum value of the γ-index is 4.3, while the mean value is 0.21. The γ-index xy slice (left) shows the γ-index distribution at z = 13 cm, which is in the peak region of the SOBP, while the other slices (center and right) are centered in x (0 cm) and y (-1.5 cm).

To obtain the cuboid, 31 energy layers from 219.0 to 277.5 MeV/u, with ~10^8^ primaries per layer, have been simulated with a total of ~1.5 × 10^9^ primary particles. In **Figure 8**, the longitudinal and lateral distribution of the SOBP are shown. The relative difference between the absorbed dose simulated by FRED and FLUKA is below 1.5%. The relative difference with respect to FLUKA predictions is within 0.2% of the total absorbed dose.

The gamma-index test has also been performed to quantify the dose distributions agreement. In **Figure 8**, the γ-index test obtained comparing FLUKA and FRED is shown. The gamma-index is strongly dependent on the statistical uncertainty, inherent to MC, which may (artificially) improve the γ pass-rate. However, it has been observed that 10^7^ primaries are enough to reduce the statistical uncertainty contribution to a negligible level.

As already observed in **Figure 8**, the dose deposited in FRED is slightly lower than the one predicted by FLUKA. However, the γ-index 2mm/3% pass-rate is 99.89% with a global cutoff of 5% of the maximum dose. This result is very good and demonstrates that FRED can be successfully used in the clinical practice.

### 3.3 Heterogeneous Materials

**Figure 9:**
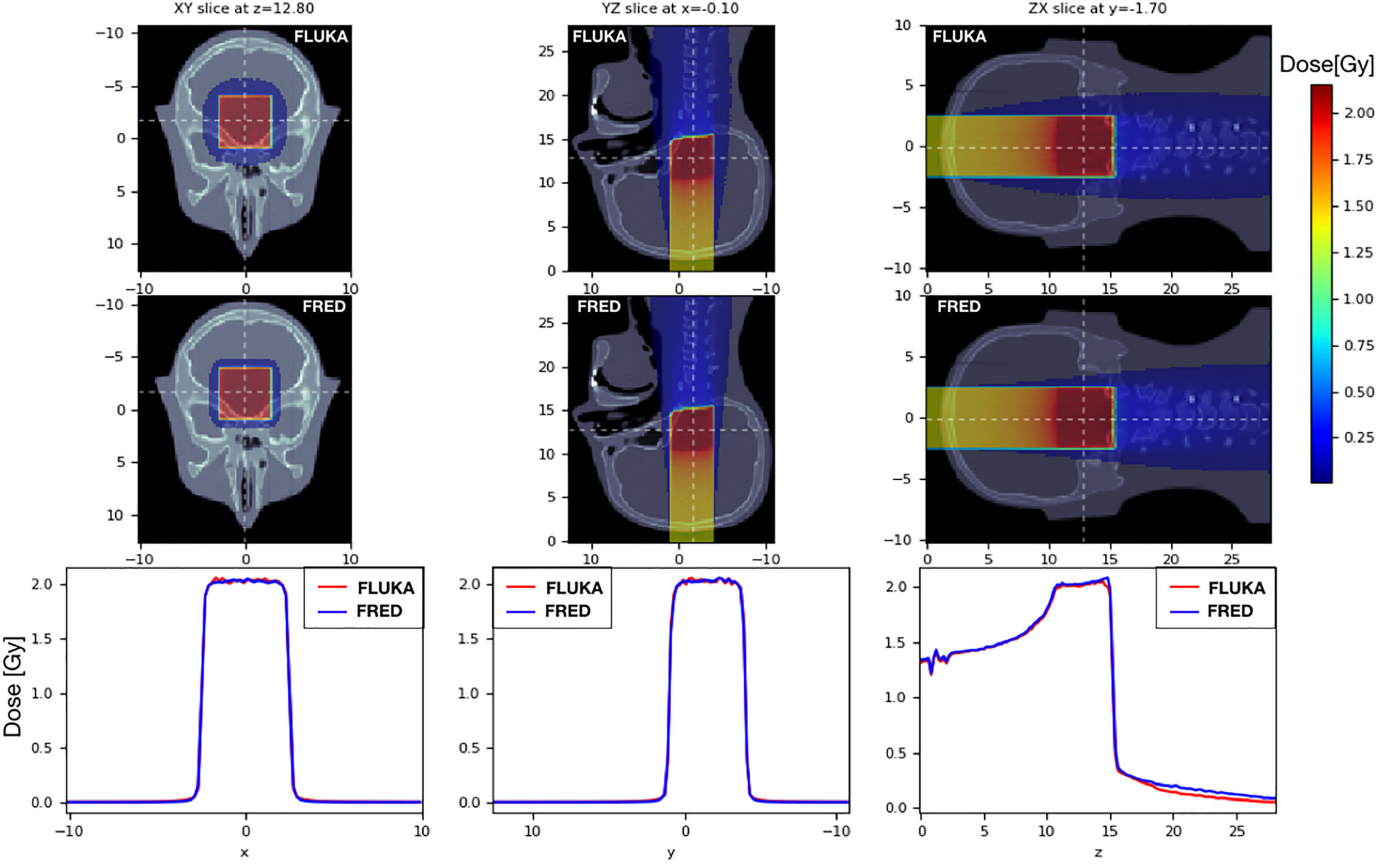
On the left the dose distribution on the XY slice at z = 12.80 cm is shown. On the center pictures, there is the dose distribution on the YZ slice centered in x. On the right, the longitudinal dose distribution on the ZX slice at z=-1.7cm is shown. The projection of the 2D figures is shown on the bottom figures. Comparison between FRED (figures on the top and blue line) and FLUKA (figures on the bottom and red line) simulations, with the same scoring grid (2 mm), and the same number of primary particles (10^6^) is shown.

To validate the FRED simulation results in heterogeneous materials, we used an anthropomorphic phantom (**Figure 9**). We delivered the same SOBP used in the previous paragraph on a head-and-neck CT, using the same calibration curve to convert HU into the material density both in FRED and in FLUKA. The CT has a voxel size of 2 mm in each direction. The 2mm/3% gamma-index between FRED and FLUKA dose distributions is 99.89% with threshold of 5%.

## 4 Performance on GPU

Once the good quality of the FRED simulation has been assessed performing a comparison with another state-of-the-art simulation software (FLUKA) the other important aspect that has to be quantified is the computation time. In **Table 5**, the time performances for different architectures are reported for the FLUKA and FRED simulations. Mono-energetic carbon beams interactions in water (target 20 cm × 20 cm × 20 cm with a 2 mm cubic voxels) were the subjects for the simulations.

**Table 5:** Computing times for different hardware architectures simulating a monoenergetic carbon ion beam at 100 (top) and 300 (bottom) MeV/u in a water target (20 cm × 20 cm× 20 cm) with 2 mm cubic voxels.

100 MeV/u				
MC	Hardware	Primary/s	*μ*s/primary
FLUKA	single CPU core	0.7 k	1400
FRED	single CPU core	4.2 k	240
FRED	single GPU card	2000 k	0.5
**300 MeV/u**				
MC	Hardware	Primary/s	*μ*s/primary
FLUKA	single CPU core	0.3 k	3000
FRED	single CPU core	3 k	300
FRED	single GPU card	2500 k	0.4

We used a motherboard with Intel Xeon E5-2687W CPU at 3,1 GHz to test the CPU performances, while we used a NVIDIA Geforce RTX 3090 for the GPU.

As it can be observed, FRED is nearly 10 times faster than FLUKA when running on the same hardware (single CPU, Intel Xeon E5-2687W at 3,1 GHz) exploiting the simplification of the implemented physics models. The tracking rate decreases with increasing energy, as expected, since a carbon ion with more energy is subject to more interactions and its average path through the medium is longer.

As shown, running on GPU (NVIDIA Geforce RTX 3090) the gain in terms of time is about three orders of magnitude with respect to single CPU execution. No significant changes to the structure of the original GPU algorithm (27) were necessary, besides the implementation of the nuclear fragmentation model for carbon described in Section 2.1. Carbon fragmentation is a relatively rare event with respect to tracing step-by-step all charged particles in a simulation. As such, the impact on the tracking rate is mostly due to the number of complete particle histories that have to be simulated per primary carbon ion. On the same GPU card and with similar geometry and scoring conditions, the typical tracking rate for a proton beam is about 5 million primary/s. The performance observed in the case of carbon ions is affected by the increased number of particles that have to be simulated. In the therapeutic energy range, such number has already been evaluated, and hence our fragmentation model generates on average 2 to 4 charged fragments per primary carbon.

## 5 Conclusion

In this contribution, we have presented a fast-MC software capable of simulating, with clinical precision, particle therapy carbon ion treatments. The nuclear fragmentation model has been developed parametrizing existent data and applying energy and angle re-scaling to estimate fragments energies in the range where data are missing. The model was based directly on experimental data, in order to ease its update whenever new or updated results will be available from experiments. For example, data from the FOOT experiment (48, 49) focusing on the study of nuclear fragmentation, will be available soon. This is the main difference between FRED and the GPU MC goCMC that has been developed starting from Geant4. In addition, by comparing the results of FRED, obtained from data, with the full-MC FLUKA, already clinically validated, there is a double check on the accuracy of the implemented model.

Results obtained when comparing FRED with FLUKA are satisfactory, especially for low energies which are the most used in PT and, in particular, for head-and-neck tumors. The relative difference between the total dose in single pencil beams in FRED and FLUKA predictions is always within 2.5% in the 100-300 MeV/u energy range. Simulating a SOBP in water the relative difference of the dose distribution is within 1.4%. For both the SOBP in water and in heterogeneous material we obtained an almost 100% pass rate for 2mm/3% gamma-index.

Beside the successful implementation of the nuclear model, capable of clinical precision when computing the absorbed dose in particle therapy conditions, FRED also achieved an impressive improvement in computing time, with respect to conventional full MC software solutions. Exploiting the parallel programming power of GPU architectures, FRED is capable of tracking millions of primary particles per second on a single GPU card. The observed gain in processing time, when comparing to the FLUKA full MC, was nearly a factor ~2000, depending on the energy of the primary beam. Using FRED in combination with GPU hardware, it is possible to process a complete treatment plan within minutes instead of days, opening the way for the use of FRED, not only for protons, but also as quality assurance tool in carbon therapy especially for the head-and-neck tumors that require lower beam energy. Comparing the time performance of FRED with the GPU MC goCMC we observed consistent results. The next step will be to compare the accuracy of FRED dose recalculation against commissioning data and commercial TPS at CNAO in order to achieve a clinical validation for carbon therapy applications.

## Data Availability Statement

The original contributions presented in the study are included in the article/supplementary material. Further inquiries can be directed to the corresponding author.

## Author Contributions

MDS: wrote the main manuscript text. MDS, VP, AnS, and GB: developed the nuclear model described in the paper. GB, MF, GT, and GF: provided FLUKA simulations used to compare the FRED model. AlS, MM, MT, and AT: provided the information about cross-sections found in literature and contributed to the interpretation of the work. PDM: provided the raster file for the SOBP and information about the clinical practice. All authors contributed to the article and approved the submitted version.

## Conflict of Interest

The authors declare that the research was conducted in the absence of any commercial or financial relationships that could be construed as a potential conflict of interest.

## Publisher’s Note

All claims expressed in this article are solely those of the authors and do not necessarily represent those of their affiliated organizations, or those of the publisher, the editors and the reviewers. Any product that may be evaluated in this article, or claim that may be made by its manufacturer, is not guaranteed or endorsed by the publisher.

## Footnotes

1 Version: 2020.0.10, using the default values provided by the HADROTHERAPY setting.
